# Are Socially Relevant Scenes Abnormally Processed in Complex Trauma-Exposed Children?

**DOI:** 10.1007/s40653-023-00549-7

**Published:** 2023-05-18

**Authors:** Clara Bertó, Belén Almansa-Tomás, Maite Ferrín, Lorenzo Livianos, Luis Rojo, María Barberá, Ana García-Blanco

**Affiliations:** 1 Department of Psychiatry and Psychology, Hospital Francesc de Borja, Gandía, Spain; 2 Neonatal Research Unit, Health Research Institute La Fe, Valencia, Spain; 3 University of Southampton, Southampton, UK; 4 ReCognition Health, London, UK; 5 Department of Psychiatry and Clinical Psychology, University and Polytechnic Hospital La Fe, Valencia, Spain; 6 Department of Medicine, University of Valencia, Valencia, Spain; 7 Department of Personality, Evaluation, and Psychological Treatment, University of Valencia, Av. Blasco Ibáñez, 21, 46010 Valencia, Spain

**Keywords:** Childhood, Maltreatment, Complex trauma, Attentional bias, Emotion

## Abstract

Abnormal attentional processes to socially relevant information may underlie behavioral dysfunctional symptoms in children exposed to a complex trauma. Attentional biases to social scenes close to real-world situations and their association with behavioral symptomatology were examined in complex trauma-exposed children. A visual dot-probe task involving neutral versus emotional (i.e., threatening, sad, or happy) scenes was applied to twenty-one maltreated children (mean age 10.43; 42.8% female; 61.1% White). These children were exposed to a complex trauma (i.e., severe, repeated, multiple, prolonged, and interpersonal) and were safeguarded in a juvenile welfare home after all parental responsibility was removed. Twenty-four comparable non-maltreated children (mean age 10.13; 29.2% female; 76% White), served as control group. All participants were at risk of social exclusion and every legal representative completed the Child Behavior Checklist (CBCL). Complex trauma-exposed children showed an attentional bias toward threatening scenes, while the control group showed an attentional bias toward sad scenes. There were no differences for happy scenes between groups. Attentional bias toward threatening scenes was associated with withdrawn symptoms in complex trauma-exposed children. Children exposed to a complex trauma show an abnormal attention to threatening social situations, which can trigger maladaptive behaviors such as withdrawn. The understanding of how complex trauma-exposed children process affective environmental information may provide new targets in the social skills interventions such as diminishing maladaptive behaviors and improving coping strategies to face threatening situations.

## Introduction

Child maltreatment has been considered a complex trauma, defined as severe, repeated, multiple, and prolonged interpersonal traumatic events that cause emotional, cognitive, and behavioral dysfunctional symptoms (Cloitre et al., 2012; DeRight, 2022; Tsur & Abu-Raiya, 2020). Complex trauma has been conceptualized as being qualitatively different from acute and single trauma in terms of its chronic and long-lasting traits. In fact, the *International Classification of Diseases*, 11th edition (ICD-11), has proposed the term Complex Posttraumatic Stress Disorder (CPTSD) as a new diagnostic category distinct from and in addition to the classic Posttraumatic Stress Disorder (PTSD), within trauma-related disorders (Brewin et al., 2017). CPTSD has been formulated to include, in addition to the core PTSD symptoms (i.e., re-experiencing the traumatic event, avoidance of traumatic reminders, and hypervigilance), disturbances in the three self-organization domains: emotion regulation, self-organization, and interpersonal functioning. In contrast to PTSD, recent data has considered disturbances of these three self-organization domains as principally responsible for the psychosocial and functional impairment characteristics of complex trauma-exposed children (Cloitre et al., 2012; D’Andrea et al., 2012). Within these self-organization disturbances, emotional dysregulation has been proposed as an essential feature, resulting in a negative self-perception and interpersonal disturbances (Bertó et al., 2017; Klein et al., 2019). Despite its importance in psychopathology development, the underlying mechanisms of emotional dysregulation in complex trauma-exposed children remain unknown. The study of emotional information processing and the role of cognitive biases in the emotional regulation observed in this population (Bertó et al., 2017; Mastorakos & Scott, 2019) may clarify this matter.

Emotional regulation develops during childhood through understanding and coping of emotions, drafting core beliefs or schemas (Shield & Cicchetti, 1997). In turn, these schemas regulate the ability to respond appropriately to the emotions of self and others (Muller et al., 2013; Powers et al., 2019). Therefore, through repeated experience, emotional dysregulation arises in complex trauma-exposed children and entails a disability to modulate affective responses against emotional stimuli, resulting in abnormal schemas (Teska & Rabin, 2017). These dysfunctional schemas are defined by a negative attribution about themselves and others (Infurna et al., 2016; Shackman et al., 2007). Taken together, these abnormal schemas result in an elevated and generalized risk perception, whereby the world is conceived as a dangerous and unpredictable place (see Blum et al., 2014). However, the exact mechanisms that explain how abnormal schemas bias the emotional information processing in the environment of complex trauma-exposed children remain under discussion.

Emotional information processing has been studied through behavioral experimental paradigms. A special mention should be made with regard to the dot-probe task, which has proved to be an excellent experimental procedure for evaluating how emotional stimuli capture attention (García-Blanco et al., 2016). To date, research provides strong evidence of an association between early exposure to maltreatment and attentional bias for negative and high-arousing emotional facial cues (i.e., threatening) (Bertó et al., 2017; Gibb et al., 2009; Pine et al., 2005). However, the direction of this abnormal negative-related processing (i.e., avoidance versus approach) is non-conclusive since literature has provided contradictory evidence. While Pine et al. (2005) reported attentional bias away from threatening faces in children with current maltreatment, Gibb et al. (2009) showed attentional bias toward threatening faces in an adult sample with documented past history of child maltreatment. Recently, Bertó et al. (2017) documented attentional biases both away from threatening faces and toward sad faces in maltreated children who were diagnosed with CPTSD. Fani et al. (2011) proposed that the period of time since the traumatic event occurred could determine the direction of attentional bias, providing a plausible explanation for the above mentioned contradictory results.

It should be noted that all available literature involving attentional capture in complex trauma-exposed children is entirely focused on facial processing (Bertó et al., 2017; Pollak & Sinha, 2002). The dot-probe task involving facial stimuli entails a valuable technique, as it allows an enhanced experimental control. Nevertheless, attentional biases from facial processing confer only a limited ecological validity and are not generalizable to real-world situations, since single stimuli in one’s environment are not often in isolation (Weierich et al., 2008). Conversely, pictures of social scenes examine emotional processing in a domain that is closer to real-world situations and offers a paradigm with higher ecological validity than facial stimuli. In fact, recent studies with other child population have documented opposite responses to threatening visual information depending on whether the stimuli were faces or scenes (see García-Blanco et al., 2017a; and García-Blanco et al., 2017b, with autism spectrum condition [ASC]). That is, whereas threatening faces were avoided (García-Blanco et al., 2017a), threatening scenes were preferably attended to (García-Blanco et al., 2017b). Thus, in complex trauma-exposed children, it may be essential to know whether the assessment of the processing of socio-emotional scenes differs from the processing of emotional facial cues, as reported in previous data (Bertó et al., 2017).

In sum, the present experiment aims to clarify the emotional processing in a homogeneous sample of currently maltreated children who were exposed to severe, repeated, multiple, and prolonged interpersonal traumatic events by using a visual dot-probe task involving neutral versus emotional (i.e., threatening, sad, or happy) scenes. First, we hypothesize that complex trauma-exposed children, relative to comparable peers, would show an abnormal attentional processing of negative information (i.e., threatening and sad scenes) (Bertó et al., 2017). Second, based on studies that have found contradictory results depending on whether the stimuli were faces or scenes (García-Blanco et al., 2017a, b), and as opposed to previous research focused on emotional facial cues (Bertó et al., 2017; Pine et al., 2005), we expect that complex trauma-exposed children would show an attentional bias toward threatening scenes. Third, according to previous data (Bertó et al., 2017) that have documented a correlation between specific attentional bias during processing of threatening faces and specific clinical features, we hypothesized that the attentional biases to negative scenes will be associated with symptoms severity.

## Method

### Participants

A total of 45 children participated in the current study. Children with documented exposure to maltreatment (maltreated group, *n* = 21; mean age 10.43; 42.8% female) were recruited from the Infant Psychiatric Unit at a tertiary hospital. Of these, 11 (52%) were Caucasian, 6 (29%) were Hispanic, 3 (14%) were African American and 1 (5%) was Asian. All participants in the clinical group experienced recent, severe, repeated, multiple, and prolonged interpersonal traumatic events. All these children experienced chronic negligence and physical abuse, and three of them also were victims of sexual abuse. Thus, all of them were safeguarded in a juvenile welfare home after their parental care was removed because of the severe circumstances of maltreatment they were exposed to. In order to obtain accurate information about previous maltreatment exposure and mental health history, social services case files were accurately examined, and social, psychological, and psychiatric non-structured interviews were conducted thoroughly. The entire complex trauma-exposed group received the diagnosis of CPTSD by the referring clinicians based on the ICD-11 proposal. Additionally to the classic PTSD symptoms (i.e., re-experiencing of the traumatic episode, avoidance of traumatic reminders, and alterations in arousal and reactivity), the complex trauma-exposed children also showed at least one CPTSD symptom in each of the three self-organization domains: 1) emotional dysregulation (excessive affective reactivity, emotional numbness, violent outbursts, suicidal thoughts, self-destructive behaviors, or dissociative states); 2) negative self-concept (increased feelings of guilt, shame, worthlessness, low self-esteem, or persistent negative beliefs about oneself); and 3) interpersonal problems (persistent difficulties building and maintaining stable relationships). The diagnosis of CPTSD was assessed by the standardized procedure conducted by Cloitre et al. (2013) using a latent profile analysis applied to a combination of two different scales: the Modified PTSD Symptom Scale-Self-Report Severity (MPSS-SR; Falsetti et al., 1993) and the Brief Symptom Inventory (BSI; Derogatis & Melisaratos, 1983).

The comparison children (non-maltreated group, *n* = 24; mean age 10.13; 29.2% female), comparable in terms of age, gender, handedness, socioeconomic status, and ethnicity, were recruited from a nearby public primary school via advertisements in the community and by word of mouth. All participants from the control group lived in their birth family’s home, and ethnically identified as 12 (50%) Caucasian, 8 (34%) Hispanic, 2 (8%) African American, and 2 (8%) Asian. Participants did not differ significantly on any socio-economic status variables and all them were at risk of social exclusion according to the *Europe 2020 strategy* (Savova, I., 2012). The legal tutor or the parents gave written consent and the study was approved by the hospital’s Bioethics Committee.

**Fig. 1:**
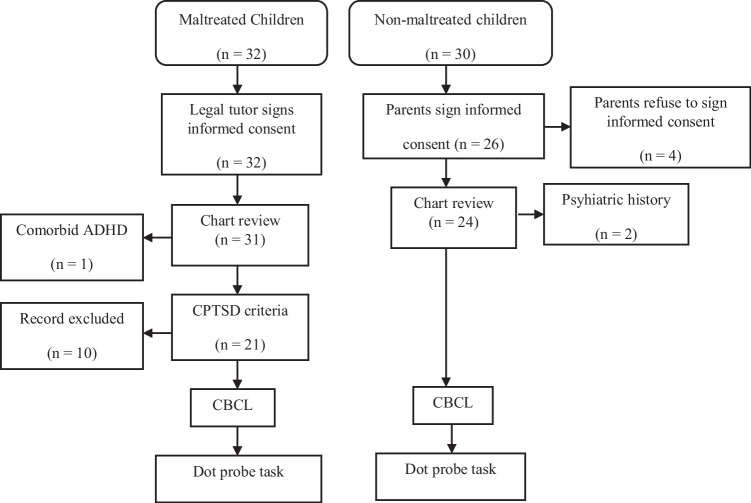
Selection process of the final sample

The exclusion criteria included relevant medical disorders, neurological diseases or a history of head trauma, visual impairment, trouble differentiating colors (e.g. color blindness), and the use of drugs that could impact cognition (i.e., corticosteroids, psychotropic medication, or antiepileptic drugs). None of these children was receiving psychotropic drugs that could affect their cognitive performance prior to the experiment. Additional psychiatric diagnosis to CPTSD was an exclusion criterion in the clinical group. Prior history of maltreatment or psychiatric disorders were also considered exclusion criteria for the control group. Figure 1 shows the selection process of the final sample.

In addition, in order to control the severity of symptomatology in the comparison children and to check for its presence in the clinical group, every legal representative completed the Child Behavior Checklist (CBCL; Achenbach & Edelbroch, 1991). The demographic and clinical data for the final sample are presented in Table 1.

**Table 1:** Sociodemographic data and clinical symptoms for each group. Data shown are means (with SD) and percentages

	Control (*n* = 24)	Maltreated (*n* = 21)	P ^a^
Female (%)	29.2%	42.8%	0.34
Race/Ethnicity, Caucasian (%)	52%	50%	0.53
Socioeconomic status ^b^	2.84 (1.31)	2.32 (1.27)	0.23
Age	10.13 (1.45)	10.43 (3.02)	0.66
CBCL scores			
Anxious/Depressed	54.88 (4.98)	64.29 (8.24)	0.000
Withdrawn/Depressed	52.92 (4.29)	74.95 (11.71)	0.000
Somatic Complains	54.08 (3.19)	61.80 (8.44)	0.000
Social Problem	53.63 (2.67)	70.32 (8.97)	0.000
Thought Problems	53.46 (5.52)	70.32 (8.97)	0.000
Attention Problems	52.87 (2.42)	69.97 (8.97)	0.000
Rule-Breaking Behavior	52.70 (4.29)	66.16 (10.91)	0.000
Aggressive Behavior	53.92 (2.76)	61.41 (6.46)	0.000
CPTSD symptoms	5.25 (3.20)	14.90 (4.98)	0.001
Re-experiencing	1.25 (1.78)	4.01 (2.88)	0.001
Avoidance	2.03 (2.03)	5.25 (2.46)	0.001
Hypervigilance	1.95 (1.28)	5.62 (2.38)	0.001
Self-organization symptoms	5.72 (3.19)	19.05 (2.87)	0.001
Affect Dysregulation	1.57 (1.67)	6.37 (1.50)	0.001
Negative Self-Concept	1.98 (1.81)	5.94 (1.80)	0.001
Interpersonal Problems	2.13 (1.60)	6.70 (1.61)	0.001

*SD* Standard deviation; *CPTSD* Complex posttraumatic stress disorder; *CBCL* Child behavior checklist

^a.^ Note, the p values correspond to Chi-squared test for sex and to t-test for the rest of variables

^b.^ The highest level of education attained by both parents was taken as indicator of socioeconomic status using a 6-point frequency rating scale ranging from 0 = for no formal qualification to 5 = for postgraduate or professional qualification

### Measures

The *Modified PTSD Symptom Scale-Self Report (MPSS-SR)* is a self-reported measure that evaluates the severity of each of the 17 PTSD symptoms outlined in the DSM-IV by using a 5-point frequency rating scale ranging from 0 = not at all to 4 = extremely. The MPSS-SR demonstrated strong psychometric properties: internal consistency coefficients ranged from 0.96 to 0.97 and inter-rater reliability was sustained by intraclass correlation coefficients from 0.21 to 0.62 (Falsetti et al., 1993; Ruglass et al., 2014).

The *Brief Symptom Inventory (BSI)* is a self-report instrument comprising 53 items that evaluate clinically relevant psychological symptoms covering nine symptom dimensions (somatization, obsession-compulsion, interpersonal sensitivity, depression, anxiety, hostility, phobic anxiety, paranoid ideation, and psychoticism). The measure evaluates how much a problem bothered or distressed a person using a 5-point Likert scale ranging from 0 = not at all to 4 = extremely. The internal consistency was sustained by alpha coefficients ranging from 0.71 to 0.82 and the test–retest reliability ranged from 0.68 to 0.91 (Derogatis & Melisaratos, 1983).

The *Child Behavior Checklist (CBCL)* is a caregiver report form identifying behavioral problems in children from 6 to 18 years of age through eight syndrome scales (anxious/depressed, withdrawn, somatic complaints social problems, thought problems, attention problems, rule-breaking behavior, and aggressive behavior). Internal consistency was supported by alpha coefficients ranging from 0.72 to 0.96, and the inter-rater and test–retest reliabilities were supported by intraclass correlation coefficients ranging from 0.93 to 1.00 (Achenbach & Edelbroch, 1991).

### Materials

In the present study, emotional stimuli serving as cues were 84 complex scenes, which had been selected from the International Affective Picture System (IAPS, Lang et al., 2005). We used the same stimuli as Kellough et al. (2008) in an experiment with distressed individuals. These stimuli were categorized as happy, neutral, sad, and threatening images,^1^ and had been rated on a 9-point scale for valence (unpleasant = 1, neutral = 5, to pleasant = 9) and arousal (calm = 1, to excitement = 9), but not for specific emotion categories. Kellough et al. (2008) conducted a pilot study with healthy individuals to identify which emotion category (threat or sadness) best described the unpleasant pictures. The valence ratings for sad (M = 2.4, SD = 0.4) and threatening images (M = 2.6, SD = 0.6) did not differ from each other (*t* < 1), while the valence for happy (M = 7.3, SD = 0.4) and neutral (M = 5.1, SD = 0.2) images were significantly different from the other three categories (*p*s < 0.001). Threatening images (M = 6.7, SD = 0.6) had greater arousal ratings than the other three categories, and neutral images (M = 2.8, SD = 0.3) had lower arousal ratings than the other three categories. However, sad images (M = 4.9, SD = 0.5) and positive images (M = 4.6, SD = 0.7) did not differ from each other in arousal ratings (*p* = 0.240).

From this set, a total of 12 happy, 12 threatening, 12 sad, and 48 neutral scenes (36 for the control and 12 for the practice trials) were selected. In each trial, two scenes were presented as cues, specifically, one emotional scene (i.e., happy, threatening, or sad) and one neutral scene. Additionally, six pairs of neutral scenes appeared as probation prior to the experiment.

### Procedure

**Fig. 2:**
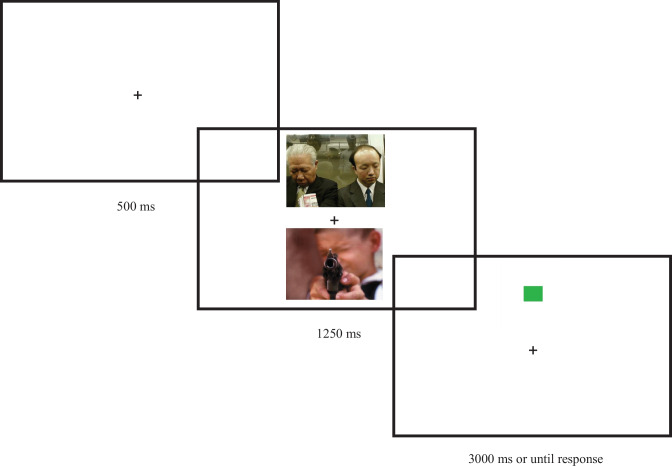
The stimulus presentation sequence under threatening condition in a neutral trial

Children were tested individually by the same clinician in a quiet sound-isolated room, at a distance of 1 m from the computer screen. DMDX software (Forster & Forster, 2003) was used to control the stimulus presentation and record the responses. In each trial, participants were instructed to look at a fixation cross ( +) for 500 ms, which appeared in the center of the screen. Then, a pair of cued scenes was presented at once at different screen locations (i.e., up and down) for 1250 ms. Following the presentation of the stimuli, a green or a red square serving as a dot appeared in the location where one of the scenes had been, either emotional (i.e., emotion trial) or neutral (i.e., neutral trial). Thereafter, the subjects were told to indicate the color of the square by pressing the “GREEN” or “RED” button, which had been painted on the keyboard. Performance was measured as time reaction and accuracy on the identification of the color. Shorter reaction times to probes appearing in the location of neutral cues signaled an attentional bias away from emotional stimuli (i.e., negative bias scores), whereas shorter reaction times to probes appearing in the location of emotional cues indicated an attentional bias toward emotional stimuli (i.e., positive bias scores). The sequence of the stimulus presentation is shown in Fig. 2.

The task comprised 9 test blocks composed of 12 trials (i.e., 4 trials for each condition: happy-neutral, threatening-neutral, and sad-neutral), which had been displayed within each block. Each pair of scenes (i.e., happy, threatening, or sad scenes paired with neutral scenes) was displayed for 3 times per task, for a total of 108 trials. Prior to the experiment, participants were trained on the task, but with different stimuli showing neutral scenes only. Hence, a total of 114 trials (108 experimental trials and 6 practice trials) were shown. The location and the category of the scene (i.e., emotional or neutral) replaced by the color square were balanced across trials, with the limitation that each category of scene was shown in each of the two positions 50% of the time and the square replaced the emotional cues 50% of the time. The presentation order of the blocks was randomized across participants. The total duration of the task was around 25–30 min.

### Data Analysis

As previously described in similar studies (Marotta et al., 2013), before evaluating the attentional bias scores, the wrong responses and trials with latencies of < 200 ms or more than 2.5 standard deviations, were discarded from further analysis after the probe response times (RTs) were calculated for the correct responses. The mean RT for each child was evaluated under each emotional valence (i.e., happy, threatening, and sad scenes). In order to control for the RTs differences between maltreated children and the non-maltreated group (e.g., 835 ms and 674 ms, respectively), the difference in proportion between the emotional (e.g., where the emotional scene was replaced by the probe) and the neutral scenes (e. g., where the neutral scene was replaced by the probe) was calculated in order to estimate the bias scores [(Mean RT neutral trials/Mean RT emotional trials*100)-100] (see Behrmann et al., 2006). Positive bias scores reflect an attentional bias toward the emotional stimulus, whereas negative bias scores reflect an attentional bias away from the emotional stimulus.

The bias score was analyzed in a 2 (Group: maltreated, control) × 3 (Valence: happy, threatening, sad) omnibus analysis of variance (ANOVA), in which Group was a between-subject factor and Valence was a within-subject factor. In case of a significant interaction, we conducted simple test effects. To test for the presence of attentional biases, the bias score was tested for the difference from zero using one-sample t-tests. Finally, bivariate correlations were conducted to check the relation of significant dot-probe bias scores and CBCL symptoms in the maltreated group.

## Results

**Fig. 3:**
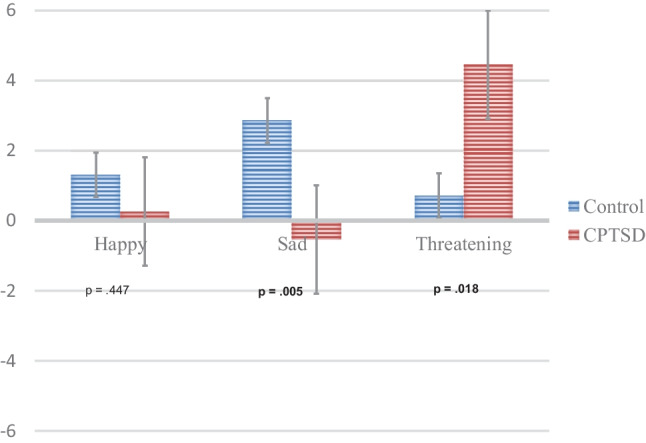
Bias scores (with SE) for the Valence x Group interaction

Preliminary analyses showed that error rates were very small in both groups (less than 2.5%), and that there were no differences between groups and conditions (all *F*s < 1). Therefore, we focused only on the RTs (see Table 2). The mean (with SE) in proportional RT differences between the neutral and the emotional conditions are shown in Fig. 3.

**Table 2:** The mean RTs (with SD) for each condition in the control and the maltreated group

	Control (*n* = 24)		Maltreated (*n* = 21)	
Valence	Emotion	Neutral	Emotion	Neutral
Happy	669 (83)	670 (80)	815 (217)	825 (219)
Sad	666 (74)	684 (74)	837 (248)	836 (254)
Threat	663 (76)	678 (80)	828 (270)	871 (303)

*RT* Response time, *SD* Standard deviation

### Overall Analysis

The ANOVA on the bias score failed to show main effects of Valence or Group (both *p*s > 0.193). More importantly, the effect of Valence differed between groups, as deduced from the significant Valence x Group interaction, *F*(2.86) = 6.06, *p* = 0.003, *η*^2^ = 0.124. This interaction revealed that, for threatening scenes, maltreated individuals showed greater bias scores than the control group*, t*(43) = 2.46, *p* = 0.018, whereas for sad scenes, the control group showed greater bias scores than maltreated individuals, *t*(48) = -2.95, *p* = 0.005. Finally, for happy scenes, there were no differences between groups, *t*(43) = 0.768, *p* = 0.45.

### Attentional Biases

Maltreated individuals showed bias scores higher than zero for threatening images, *t*(20) = 3.47, *p* = 0.002, but not for happy or sad scenes (both *p*s > 0.208). The control group showed bias scores higher than zero for sad images *t*(23) = 4.59, *p* < 0.001, but not for happy and threatening scenes (both *p*s > 0.424).

### Association Between CBCL Symptoms and Significant Bias Scores

To further examine the association between the attentional bias toward threatening images and the behavioral syndromes in maltreated children, we computed the Pearson correlation coefficient between the threat-related bias score and the CBCL scores. The CBCL Withdrawal subscale had a positive relationship with threatening scores in maltreated children (*r* = 0.473, *p* = 0.030). That is, higher scores on withdrawal were associated with higher bias to threatening scenes. No other CBCL subscales correlations were significant (all *p*s > 0.15).

## Discussion

The current study aimed to examine the attentional processing of socio-emotional (i.e., threatening, sad, and happy) scenes by means of a dot-probe task in a sample of maltreated children recently exposed to, severe, repeated, multiple, and prolonged interpersonal traumatic events. The main finding was that complex trauma-exposed children showed a different emotional processing of negative information relative to the control group, depending on the level of arousal. Moreover, according to our second hypothesis, complex trauma-exposed children showed an attentional bias toward threatening scenes, which is opposite to the bias away from threatening faces found in previous studies (Bertó et al., 2017). A final relevant finding was the correlation between attentional biases and clinical symptoms, which showed that the higher the attentional bias toward threatening scenes in complex trauma-exposed children, the higher the withdrawal symptoms.

As predicted in our first hypothesis, relative to the control group, complex trauma-exposed children displayed an abnormal processing of negative information, which was modulated by the arousal of the emotional scene. That is, whereas complex trauma-exposed children showed an attentional bias toward threatening scenes, they did not show the typical attentional bias toward sad scenes displayed by the control group (García-Blanco et al., 2017b; Kisley et al., 2007). Interpretation of the attentional bias toward threatening social scenes can be placed in the context of previous data (Powers et al., 2019; Shield & Cicchetti, 1998), which suggests that maltreated children, in response to their current exposure to complex trauma, develop a biased perception of the environment when threatening situations occur. According to this approach, preferential attention to threatening environmental cues may result initially as adaptive for children, for example, to develop an increased vigilance to detect anger, as this may facilitate attempts to avoid dangerous and unpredictable situations (Cicchetti & Toth, 1995). However, whereas the attentional preference of threatening stimuli may be an adaptive behavior in the short term, the maintenance of this preference may result a maladaptive behavior in the long term. For instance, cumulative research has suggested that this selective attention for threatening social cues after exposure to trauma leads to a hypervigilant state, which could play a role in maintaining CPTSD symptoms (Cusmano, 2016; Mastorakos & Scott, 2019; Pollak et al., 1997). In addition, the hypervigilance to a threatening social situation may contribute to an inability to re-direct the attention away from related cues (Cusmano, 2016). Taken together, the difficulty to disengage from threatening situations may trigger negative thoughts and feelings (Kimble et al., 2014), which maintain their focus on distressing stimuli (Shield & Cicchetti, 1997) and contribute to the maintenance of abnormal schemas in complex trauma-exposed children.

In relation to the opposite attentional pattern observed between faces and scenes, whereas the current study revealed an attentional bias toward threatening scenes in a homogeneous sample of maltreated children (i.e., recently exposed to severe, repeated, multiple, and prolonged interpersonal traumatic situations), previous studies with a similar sample had reported an attentional bias away from threatening faces (Bertó et al., 2017; Pine et al., 2005). These apparently contradictory data between faces and scenes are comparable with previous studies focused on ASC children (see García-Blanco et al., 2017a [for an attentional bias away from angry faces] and see García-Blanco et al., 2017b [for an attentional bias toward threatening scenes]). Cumulative research may account for this opposite pattern. Faces are considered as simple stimuli and entail low-level brain processing (McCrory et al., 2013; Tottenham et al., 2011). In line with this finding, complex trauma-exposed children may display difficulties in detecting threatening faces and reacting appropriately (Haxby et al., 2002). In contrast, scenes are considered as complex stimuli closer to real-world social situations, which are linked to high-level brain processing as a regulatory mechanism of low-level brain areas (Heitmann et al., 2016). A poor disengagement from threatening environments may also hinder an appropriate reaction (Mueller-Pfeiffer et al., 2010). Therefore, these findings can be understood under the theoretical approach that posits that maltreated children perceive the world as an unpredictable and dangerous place (Blum et al., 2014). That is, the lack of attention to angry faces and the engagement of threatening situations can increase their risk perception, their negative self-perception, and a biased perception of others (Infurna et al., 2016; Shackman et al., 2007).

Notably, when the association between clinical symptoms and attentional biases were examined, the higher the attentional bias toward threatening scenes, the more significant the withdrawal symptoms in complex trauma-exposed children were found. A plausible explanation of this finding can also be placed in the context of previous research focused on maltreated children. According to Pollak et al. (1997), the lack of disengagement with threatening environments can result in withdrawal behaviors, due to the absence of coping strategies (Shield & Cicchetti, 1998). Consequently, the maintenance of this attentional bias and the withdrawal reaction can be associated with an overestimation of stimuli as threatening and perhaps mistaken judgments regarding ambiguous or unusual social situations (Pollak & Klister, 2002). Thus, maltreated children may experience an exaggerated need to defend themselves from erroneous perceived social threats in a maladaptive manner (Gladwin, 2017; Tsur & Abu-Raiya, 2020), responding through withdrawal. In turn, complex trauma-exposed children may show withdrawal symptoms, which eventually would lead to an abnormal attention to threatening social situations.

### Limitations

There are a number of limitations to the current study that should be mentioned. Firstly, this research is a cross-sectional study, which constrains temporal and causal inferences between complex trauma and the abnormal processing of threats. Thus, longitudinal studies are required to address issues of causality and whether observed findings remain over time, which would provide valuable data about the underlying pathways of complex trauma exposure and its clinical presentation. Secondly, because the study examined complex trauma-exposed children diagnosed from CPTSD based on the ICD-11 proposal, the degree to which threat-related biases are associated with CPTSD as opposed to child maltreatment remains unclear. Thus, further studies that compare CPTSD children to maltreated children without CPTSD should be conducted. Thirdly, despite our sample in the clinical group was reasonably homogeneous in terms of complex trauma exposure to avoid potential confounders (i.e., the whole maltreated group was recently exposed to severe, repeated, multiple, prolonged and interpersonal traumatic events), the final sample size was relatively small. Finally, the potential recruitment bias may constitute another limitation. Although the children were comparable in terms of relevant demographic variables (i.e., age, gender, handedness, socioeconomic status, and ethnicity), the partial recruitment of the control group from a public school might represent a cohort-effect bias. Nevertheless, the main studied variable (i.e., the valence of social scenes) was a within-subject factor.

### Therapeutic, Diversity-Related and Future Research Implications

In conclusion, complex trauma exposure is associated with an abnormal processing of threats. Furthermore, this attentional bias toward threatening scenes may underlie concomitant clinical patterns in terms of withdrawal symptoms. From an applied viewpoint, the attentional bias toward threatening scenes may explain the behavioral symptoms of complex trauma-exposed children during real-world situations. That is, they may learn that withdrawal is an acceptable way of responding to threats and managing conflict. The findings add novel information to the field´s understanding of the etiology of emotional dysregulation in complex trauma-exposed children, resulting in major implications for novel therapeutic interventions, diversity-related implications, and future investigation. With regard to therapeutic implications, if abnormal emotional processing has been suggested to contribute to the maintenance of behavioral symptoms, Attention Bias Modification Training (ABMT) can be a new target of clinical treatment for reducing child emotional and behavioral problems (Coventry et al., 2020; Karatzias et al., 2019; Mastorakos & Scott, 2019). Related to diversity implications, although our sample was highly homogeneous in terms of complex trauma exposure, it was culturally and ethnically diverse, which may play an important role in the generalization of findings to groups outside the present sample. Regarding research implications, future dot-probe studies may benefit from the inclusion of eye-tracking technologies that allow a more fine-grained (in terms of location and time-monitoring) analysis of attentional patterns (Garner et al., 2016). Moreover, longitudinal studies are required in order to prove whether withdrawal symptoms improve following a modification of this threat-related attentional bias in complex trauma-exposed children. Finally, a follow-up of the present sample may be performed in order to test if attentional bias toward threatening scenes in complex trauma-exposed children represents a risk marker in association with vulnerability to future psychiatric disorders (Briggs-Gowan et al, 2015).

## Footnotes

1 The following IAPS images were used: sad – 2141, 2205, 2276, 2455, 2700, 2703, 2799, 2900, 3230, 9220, 9421, and 9530; threat – 1120, 1300, 2811, 3500, 6260, 6312, 6313, 6350, 6510, 6560, 6562, and 6821; happy – 1340, 2091, 2165, 2208, 2224, 2299, 2339, 2340, 2501, 4599, 4700, and 8461; neutral – 2038, 2102, 2190, 2191, 2215, 2235, 2393, 2396, 2397, 2487, 2514, 2516, 2745, 2850, 2880, 5500, 5390, 5731, 5740, 7000, 7004, 7009, 7010, 7041, 7053, 7080, 7090, 7100, 7185, 7187, 7235, 7547, 7493, 7496, 7550, and 7950; neutral practice –2840, 2870, 5130, 7031, 5390, 5395, 5661, 5900, 6000, 6150, 8311, and 9070.
